# Supplementary material for: Genetic associations with carotid intima-media thickness link to atherosclerosis with sex-specific effects in sub-Saharan Africans
Source: Nat Commun. 2022 Feb 14;13:855. doi: 10.1038/s41467-022-28276-x (PMC8844072; doi:10.1038/s41467-022-28276-x)
Supplement: Supplementary file 1 — Supplementary Information [file 41467_2022_28276_MOESM1_ESM.pdf]

Genetic associations with carotid intima-media thickness link to atherosclerosis with sex-specific effects in sub-Saharan Africans

Palwende Romuald Boua[1,2,3*], Jean-Tristan Brandenburg[2] , Ananyo Choudhury[2], Hermann Sorgho[1], Engelbert A. Nonterah[4,5], Godfred Agongo[4,6], Gershim Asiki[7], Lisa Micklesfield[8], Solomon Choma[9], Francesc Xavier Gómez-Olivé[10], Scott Hazelhurst[11], Halidou Tinto[1], Nigel J. Crowther[12], Christopher G. Mathew[2,3,13], Michèle Ramsay[2,3*] as members of AWI-Gen Study and the H3Africa Consortium

[1]Clinical Research Unit of Nanoro, Institut de Recherche en Sciences de la Santé, Burkina Faso
[2] Sydney Brenner Institute for Molecular Bioscience, Faculty of Health Sciences, University of the Witwatersrand, Johannesburg, South Africa
[3] Division of Human Genetics, National Health Laboratory Service and School of Pathology, Faculty of Health Sciences, University of the Witwatersrand, South Africa
[4] Navrongo Health Research Centre, Ghana Health Service, Navrongo, Ghana
[5] Julius Global Health, Julius Centre for Health Sciences and Primary Care, University Medical Centre, Utrecht University, The Netherlands
[6] C.K. Tedam University of Technology and Applied Sciences, Navrongo, Ghana
[7]African Population and Health Research Center, Nairobi, Kenya
[8] MRC/Wits Developmental Pathways for Health Research Unit, Faculty of Health Sciences, University of the Witwatersrand, Johannesburg, South Africa
[9] Department of Pathology and Medical Sciences, University of Limpopo, South Africa
[10] MRC/Wits Rural Public Health and Health Transitions Research Unit (Agincourt), School of Public Health, Faculty of Health Sciences, University of the Witwatersrand, Johannesburg, South Africa
[11] School of Electrical and Information Engineering, University of the Witwatersrand, Johannesburg, South Africa.
[12]Department of Chemical Pathology, National Health Laboratory Service, Faculty of Health Sciences, University of the Witwatersrand, Johannesburg, South Africa
[13] Department of Medical and Molecular Genetics, Faculty of Life Sciences and Medicine, King's College London, London, United Kingdom

* Corresponding authors: Palwende Romuald Boua (romyboua@gmail.com) and Michèle Ramsay (michele.ramsay@wits.ac.za)

Supplementary Note 1

1. F-box and leucine rich repeat protein 17 (*FBXL17*)

Our study identified rs552690895 (p = 2.5E-08) in *FBXL17* in the combined set. F-box and leucine rich repeat protein 17 (*FBXL17*) is characterized by an approximately 40-amino acid F-box motif. SCF complexes are formed by *SKP1* (S-phase kinase-associated protein 1), cullin (*CUL1*), and F-box proteins, and act as protein-ubiquitin ligases. F-box proteins interact with SKP1 through the F-box, and they interact with ubiquitination targets through other protein interaction domains (1). Evidence suggest that the SCF is a key complex in the ubiquitine-proteasome system (UPS) that is involved in 70.0-90.0% of protein degradation processes (2). It has been found that protein degradation by the UPS play a central role in cardiovascular physiology and disease: from endothelial function, the cell cycle, atherosclerosis, myocardial ischaemia, cardiac hypertrophy, inherited cardiomyopathies, and heart failure (3–7). A GWAS in Lithuanian families found that variants in *FBXL17* were associated with coronary heart diseases (8). Other reports on genetic associations for *FBXL17* include studies for educational attainment and mathematical ability (9), intelligence (10), and pulse pressure (11).

2. Signal regulatory protein alpha (*SIRPA*)

Our combined analysis identified also genetic association of rs6045318 (p = 4.7E-08) with cIMT in the *SIRPA* gene. Signal regulatory protein alpha (*SIRPA*) is a regulatory membrane glycoprotein from the SIRP family, which inhibits the cytoskeleton-intensive process of phagocytosis by the macrophage. *SIRPA* is activate cancer cells (through high expression of CD47) and upregulated *SIRPA* inhibits macrophage-mediated destruction. This mediation of phagocytosis and polarization of macrophages is important in the pathophysiology of atherosclerosis (12). There is evidence that *SIRPA* is involved in discrete stages of cardiovascular cell lineage differentiation (13) and that defects in the gene (knock out) reduces atherosclerosis in mice (14). *SIRPA* expression has been found as a signature of inflamed atherosclerotic plaque (15). Previous reports on GWAS studies associate *SIRPA* with blood protein (16,17), and the percentage of basophil in granulocytes (18).

3. Sorting nexin 29 (*SNX29*)

On the chromosome 16, rs147978408 (p = 6.3E-09) was the top cIMT associated variant in *SNX29* for the male-specific analysis. The sorting nexin (*SNX*) family is a diverse group of cytoplasmic- and membrane-associated phosphoinositide-binding proteins that play pivotal roles in the regulation of protein trafficking. *SNX* gene variants are associated with CVDs, and dysfunction of the *SNX* pathway is involved in several forms of cardiovascular disease (CVD) (19). In a study of genes that regulate smooth muscle cell differentiation and disease risk, *SNX29* was involved in pathways for occlusion of blood vessels and atherosclerosis (20). Ito and collaborators identified sex-dependent differentially methylated regions close to *SNX29* in mouse liver and found that this methylation status was influenced by testosterone and contributed to sex-dimorphic chromatin decondensation (21). This might explain the

sex-specific effect observed in our study. A study in children with sickle cell disease, identified *SNX29* variants as suggestive of association with systolic blood pressure (22). Also, variants in *SNX29* were found in suggestive association with subcutaneous adipose tissue in women (Sung *et al*. 2016). In patients with pulmonary arterial hypertension, *SNX29* variation was reported for differential responses to vasodilator treatment (24). Further GWAS analysis stratified by hypertensive status showed that the association was driven by the hypertensive group (effect three times higher in hypertensives compared to the non-hypertensives), therefore demonstrating that the association of *SNX29* with cIMT might be mediated by the vascular remodeling caused by hypertension. GWA studies reported *SNX29* variants for association with educational attainment, mathematical ability and cognitive function measurement (Lee *et al*. 2018), intelligence (10,25), bone mineral density (26), and smoking (Liu *et al*. 2019).

4. Mitogen-activated protein kinase kinase kinase 7 (*MAP3K7*)

In the male-specific analysis, we found rs284509 (p=5.3E-08) in *MAP3K7* region on chromosome 6 to be associated with cIMT. Mitogen-activated protein kinase kinase kinase 7 (*MAP3K7*) also called *TAK1* encodes a serine/threonine protein kinase family member, with a central role as regulator of cell death. Because of its role in kinase pathway, and regulation of transforming growth factor beta (TGF-β), *MAP3K7* plays a role in growth inhibition in vascular smooth muscle cells and can be atheroprotective or atherogenic (28). More biological evidence of the contribution of *MAP3K7* to atherosclerosis is through its regulation by micro-RNAs (29,30). In a study of women receiving hormone replacement therapy, variants in *MAP4K4,* a gene targeting *MAP3K7* (31) were associated to cIMT (32). The sex-specific association observed might be related to the fact that *MAP3K7IP3* (located on the X chromosome), which is known to form a ternary complex with *MAP3K7* in response to inflammatory stimuli, has shown sex-differential expression in ischemic stroke (33,34). In a study on expression of androgen-modulated micro-RNAs, it has been reported that *MAP3K7* was a target of mmu-miR-467h and mmu-miR-669i in the angiogenesis and transforming growth factor beta receptor signalling pathways (35). Despite biological relevance to atherosclerosis, GWA studies reported variants in gene region to be associated with cancer progression (36), anti-TNF response in rheumatoid arthritis (37), attention deficit hyperactivity disorder (38), adolescent idiopathic scoliosis (Liu *et al*. 2018), and sporadic amyotrophic lateral sclerosis (Xie *et al*. 2014). Our study is the first to report *MAP3K7* association with a CVD phenotype.

5. La-related protein 6 (*LARP6*)

LARP6 (La-related protein 6) is a ribonucleoprotein domain family member 6. Studies showed that it has a role in collagen regulation by targeting mRNA encoding Type I collagen (Cai *et al*. 2010; Zhang and Stefanovic 2016; Glenn, Wang, and Schwartz 2009; Stefanovic *et al*. 2019). Sukhanov and collaborators found that *IGF1R* deficiency downregulated collagen mRNA-binding protein *LARP6* and vascular collagen, and showed an atheroprotective effect (45,46). Collagen is a hallmark of atherosclerotic plaque stability, thus alteration of the collagen balance may lead to an instability of atherosclerotic lesions, and therefore promote plaque formation and rupture (15,47). In the Taiwanese population, the *LARP6* locus was found to be associated with coronary artery disease (48). In European ancestry populations,

LARP6 was found associated with insulin measurement (49). However, Mendelian randomization for cIMT found that despite the limited effects of proinsulin-increasing SNP scores on cIMT, proinsulin was unlikely to have causal effects on cIMT (50). Myocardial gene expression in non-ischemic human heart failure found *LARP6* to be differentially expressed between men and women (1.36 fold) (51). The female-specific effect of the loci may find its explanation in the enhancer function of rs78172571 in high LD with rs150840489 (the top SNP associated in our female-specific) on *THAP10* gene (FDR = 2.03E-17) known to be regulated by oestrogen.

6. Prokineticin 1 (*PROK1*)

Prokineticin 1 (*PROK1*), also called endocrine gland derived vascular endothelial growth factor (*EG-VEGF*), is a specific placental angiogenic factor which play a role in the control of normal (e.g endometrial decidualization) and pathological placental angiogenesis (52). It is involved in pathologies such as recurrent pregnancy loss, gestational trophoblastic diseases, foetal growth restriction, and preeclampsia (53–57). The gene is known to be predominantly expressed in the steroidogenic glands, such as ovary, testis, and adrenal cortex, and is often complementary to the expression of vascular endothelial growth factor (*VEGF*), suggesting that these molecules function in a coordinated manner. The function and particular pattern of this gene's activity might explain why we identified the locus only in our female-specific analysis. Our study is the first to report *PROK1* for any trait in a GWAS.

7. Caldesmon 1 (*CALD1*)

Our gene-based analysis identified caldesmon 1 significantly associated with cIMT in our combined set led by rs7781307 (p = 2.1E-06) on 7q33. Caldesmon 1 is calmodulin binding protein encoding for a calmodulin-and actin-binding protein that play a major role in the regulation of smooth muscle contraction, cell migration and cell invasion (58). *CALD1* was identified as key gene in the "regulation of actin cytoskeleton" module from protein-protein interaction network resulting from a bioinformatics analysis of key pathways and genes in advanced coronary atherosclerosis (59). A study screening for keys genes for abdominal aortic aneurysm found that *CALD1* was leading a KEGG enrichment signal pathways (Vascular smooth muscle contraction) of differential expressed genes (DEGs) (60). Underexpression of *CALD1* was found to be a key feature of calcification of vascular smooth muscle cells from atherosclerotic plaque (15,61,62). Additionally, studies on epigenetic modifications reported *CALD1* to exhibit differential methylation in atherosclerosis (63–65). Previous GWAS reported CALD1 for phenotypes such as Lung function (FEV1/FVC) (66), Response to paliperidone in schizophrenia (PANSS score) (Li *et al*. 2017), Attention deficit hyperactivity disorder symptom score (68), Diverticular disease (69)

8. Fms-related tyrosine kinase (*FLT4*)

FLT4 or Vascular endothelial growth factor receptor 3 (*VEGFR3*) is a major signalling protein involved in angiogenesis, vasculogenesis and maintenance of the endothelium. By acting as receptor to VEGFC and VEGFD, it plays an essential role in lymphangiogenesis in adults and in the development cardiovascular system during embryonic phase. Defect and/or downregulation of *VEGR3* was found to lead to cardiovascular failure in embryonic stage

and to higher mortality after myocardial infarction in mice models (70,71). Biological studies have highlighted the role of *FLT4* in atherosclerosis in major pathological processes. The gene has been reported to be involved in plaque instability by two process: the mediation of monocytes/macrophages apoptosis and consequently alteration plaque stability (72) ; and the modulation of vascular remodelling and shear stress resulting in plaques haemorrhages and calcification in carotids (73–75). Our study is the first to report association of *FLT4* locus (rs112967731, p = 5.7E-07, female-specific) with cIMT or any cardiovascular phenotype in GWAS studies. Previous studies reported the locus for association with folic acid measurement (76) and blood protein measurements (16,17).

References

1. Jin J, Cardozo T, Lovering RC, Elledge SJ, Pagano M, Harper WJ. Systematic analysis and nomenclature of mammalian F-box proteins. Genes Dev [Internet]. 2004;18(1):2573–80. Available from: http://dx.doi.org/10.1101/gad.1255304
2. Depre C, Powell SR, Wang X. The role of the ubiquitin-proteasome pathway in cardiovascular disease. Cardiovasc Res. 2010;85(2):251–2.
3. Herrmann J, Lerman LO, Lerman A. On to the road to degradation: Atherosclerosis and the proteasome. Cardiovasc Res. 2010;85(2):291–302.
4. Fasanaro P, Capogrossi MC, Martelli F. Regulation of the endothelial cell cycle by the ubiquitin-proteasome system. Cardiovasc Res. 2010;85(2):272–80.
5. Stangl K, Stangl V. The ubiquitin-proteasome pathway and endothelial (dys)function. Cardiovasc Res. 2010;85(2):281–90.
6. Powell SR, Divald A. The ubiquitin-proteasome system in myocardial ischaemia and preconditioning. Cardiovasc Res. 2010;85(2):303–11.
7. Yu X, Kem DC. Proteasome inhibition during myocardial infarction. Cardiovasc Res. 2010;85(2):312–20.
8. Domarkiene I, Pranculis A, Germanas Š, Jakaitiene A, Vitkus D, Dženkevičiute V, et al. RTN4 and FBXL17 genes are associated with coronary heart disease in genome-wide association analysis of Lithuanian families. Balk J Med Genet. 2013;16(2):17–22.
9. Lee JJ, Wedow R, Okbay A, Kong E, Maghzian O, Zacher M, et al. Gene discovery and polygenic prediction from a genome-wide association study of educational attainment in 1.1 million individuals. Nat Genet. 2018;50(8):1112–21.
10. Davies G, Lam M, Harris SE, Trampush JW, Luciano M, Hill WD, et al. Study of 300,486 individuals identifies 148 independent genetic loci influencing general cognitive function. Nat Commun. 2018;9(1):1–16.
11. Evangelou E, Warren HR, Mosen-Ansorena D, Mifsud B, Pazoki R, Gao H, et al. Genetic analysis of over 1 million people identifies 535 new loci associated with blood pressure traits. Nat Genet. 2018;50(10):1412–25.
12. Chen W, Li X, Wang J, Song N, Zhu A, Jia L. miR-378a Modulates Macrophage Phagocytosis and Differentiation through Targeting CD47-SIRPα Axis in Atherosclerosis. Scand J Immunol. 2019;90(e12766):1–10.
13. Skelton RJP, Costa M, Anderson DJ, Bruveris F, Finnin BW, Koutsis K, et al. SIRPA, VCAM1 and CD34 identify discrete lineages during early human cardiovascular development. Stem Cell Res [Internet]. 2014;13(1):172–9. Available from: http://dx.doi.org/10.1016/j.scr.2014.04.016
14. Szilagyi K, Gijbels MJJ, Pottgens C, van Gorp PJ, Binder C, Kraal G, et al. Defective signal regulatory protein alpha (sirpa) signaling reduces atherosclerosis in mice. Atherosclerosis [Internet]. 2014;235(2):e91. Available from: http://dx.doi.org/10.1016/j.atherosclerosis.2014.05.242
15. Puig O, Yuan J, Stepaniants S, Zieba R, Zycband E, Morris M, et al. A gene expression signature that classifies human atherosclerotic plaque by relative inflammation status. Circ Cardiovasc Genet. 2011;4(6):595–604.
16. Emilsson V, Ilkov M, Lamb JR, Finkel N, Gudmundsson EF, Pitts R, et al. Co-regulatory networks of human serum proteins link genetics to disease. Science (80-). 2018;361(6404):769–73.
17. Sun BB, Maranville JC, Peters JE, Stacey D, Staley JR, Blackshaw J, et al. Genomic atlas

of the human plasma proteome. Nature [Internet]. 2018;558(7708):73–9. Available from: http://dx.doi.org/10.1038/s41586-018-0175-2
18. Astle WJ, Elding H, Jiang T, Allen D, Ruklisa D, Mann AL, et al. The Allelic Landscape of Human Blood Cell Trait Variation and Links to Common Complex Disease. Cell. 2016;167(5):1415-1429.e19.
19. Yang J, Villar VAM, Rozyyev S, Jose PA, Zeng C. The emerging role of sorting nexins in cardiovascular diseases. Clin Sci. 2019;133(5):723–37.
20. Iyer D, Zhao Q, Wirka R, Naravane A, Nguyen T, Liu B, et al. Coronary artery disease genes SMAD3 and TCF21 promote opposing interactive genetic programs that regulate smooth muscle cell differentiation and disease risk. PLoS Genet. 2018;14(10):1–28.
21. Ito S, Hirabayashi K, Moriishi K, Matsui Y, Moriya K, Koike K, et al. Novel sex-dependent differentially methylated regions are demethylated in adult male mouse livers. Biochem Biophys Res Commun [Internet]. 2015;462(4):332–8. Available from: http://dx.doi.org/10.1016/j.bbrc.2015.04.137
22. Bhatnagar P, Barron-Casella E, Bean CJ, Milton JN, Baldwin CT, Steinberg MH, et al. Genome-Wide Meta-Analysis of Systolic Blood Pressure in Children with Sickle Cell Disease. PLoS One. 2013;8(9):1–11.
23. Sung YJ, Pérusse L, Sarzynski MA, Fornage M, Sidney S, Sternfeld B, et al. Genome-wide association studies suggest sex-specific loci associated with abdominal and visceral fat. Int J Obes. 2016;40(4):662–74.
24. Karnes JH, Batai K, Arora A, Kaye J, Steiner H, Nair V, et al. Abstract 24015: Genome-Wide Association Study of Vasodilator Response in Pulmonary Arterial Hypertension. Circulation [Internet]. 2017 Nov 14;136(suppl_1):A24015–A24015. Available from: https://doi.org/10.1161/circ.136.suppl_1.24015
25. Savage JE, Jansen PR, Stringer S, Watanabe K, Bryois J, Leeuw CA De, et al. Genome-wide association meta-analysis in 269,867 individuals identifies new genetic and functional links to intelligence. Nat Genet. 2018;50(7):912–9.
26. Morris JA, Kemp JP, Youlten SE, Laurent L, Logan JG, Chai RC, et al. An atlas of genetic influences on osteoporosis in humans and mice. Nat Genet. 2019;51(2):258–66.
27. Liu M, Jiang Y, Wedow R, Li Y, Brazel DM, Chen F, et al. Association studies of up to 1.2 million individuals yield new insights into the genetic etiology of tobacco and alcohol use. Nat Genet. 2019;51(2):237–44.
28. Toma I, MacCaffrey TA. Transforming growth factor-β and atherosclerosis: interwoven atherogenic and atheroprotective aspects. Cell Tissue Res. 2012;118(24):155–75.
29. Paone S, Baxter AA, Hulett MD, Poon IKH. Endothelial cell apoptosis and the role of endothelial cell-derived extracellular vesicles in the progression of atherosclerosis. Cell Mol Life Sci [Internet]. 2019;76(6):1093–106. Available from: https://doi.org/10.1007/s00018-018-2983-9
30. Njock M-S, Cheng HS, Dang LT, Nazari-Jahantigh M, Lau AC, Boudreau E, et al. Endothelial cells suppress monocyte activation through secretion of extracellular vesicles containing antiinflammatory microRNAs. Blood. 2015;125(20):3202–12.
31. Xie M, Zhang D, Wang SC, Sano M, Wang X, Pocius JS, et al. Abstract 1949: The Protein Kinase MAP4K4 Is Activated in Failing Human Hearts and Mediates Cardiomyocyte Apoptosis in Experimental Models, in vitro and in vivo. Circulation [Internet]. 2007 Oct 16;116(suppl_16):II_420-II_420. Available from:

https://doi.org/10.1161/circ.116.suppl_16.II_420
32. Miller VM, Petterson TM, Jeavons EN, Lnu AS, Rider DN, Heit JA, et al. Genetic polymorphisms associated with carotid artery intima-media thickness and coronary artery calcification in women of the Kronos Early Estrogen Prevention Study. Physiol Genomics [Internet]. 2013;45(2):79–88. Available from: http://www.ncbi.nlm.nih.gov/pubmed/23188791%5Cnhttp://physiolgenomics.physiology.org/content/physiolgenomics/45/2/79.full.pdf
33. Stamova B, Tian Y, Jickling G, Bushnell C, Zhan X, Liu D, et al. The X-chromosome has a different pattern of gene expression in women compared with men with ischemic stroke. Stroke. 2012;43(2):326–34.
34. Rocha DM, Caldas AP, Oliveira LL, Bressan J, Hermsdorff HH. Saturated fatty acids trigger TLR4-mediated inflammatory response. Atherosclerosis [Internet]. 2016;244:211–5. Available from: http://dx.doi.org/10.1016/j.atherosclerosis.2015.11.015
35. Bouhaddioui W, Provost PR, Tremblay Y. Expression profile of androgen-modulated microRNAs in the fetal murine lung. Biol Sex Differ [Internet]. 2016;7(1):1–13. Available from: http://dx.doi.org/10.1186/s13293-016-0072-z
36. Penney ME, Parfrey PS, Savas S, Yilmaz YE. A genome-wide association study identifies single nucleotide polymorphisms associated with time-to-metastasis in colorectal cancer. BMC Cancer [Internet]. 2019;19(1):133. Available from: http://ovidsp.ovid.com/ovidweb.cgi?T=JS&PAGE=reference&D=emexb&NEWS=N&AN=626323548
37. Honne K, Hallgrímsdóttir I, Wu C, Sebro R, Jewell NP, Sakurai T, et al. A longitudinal genome-wide association study of anti-tumor necrosis factor response among Japanese patients with rheumatoid arthritis. Arthritis Res Ther [Internet]. 2016;18(1):1–10. Available from: http://dx.doi.org/10.1186/s13075-016-0920-6
38. Lasky-Su J, Anney R, Neale BM, Franke B, Zhou K, Maller JB, et al. Genome-wide association scan of attention deficit hyperactivity disorder. Am J Med Genet Part B Neuropsychiatr Genet. 2008;147(8):1337–44.
39. Liu J, Zhou Y, Liu S, Song X, Yang XZ, Fan Y, et al. The coexistence of copy number variations (CNVs) and single nucleotide polymorphisms (SNPs) at a locus can result in distorted calculations of the significance in associating SNPs to disease. Hum Genet [Internet]. 2018;137(6–7):553–67. Available from: http://dx.doi.org/10.1007/s00439-018-1910-3
40. Xie T, Deng L, Mei P, Zhou Y, Wang B, Zhang J, et al. A genome-wide association study combining pathway analysis for typical sporadic amyotrophic lateral sclerosis in Chinese Han populations. Neurobiol Aging [Internet]. 2014;35(7):1778.e9-1778.e23. Available from: http://dx.doi.org/10.1016/j.neurobiolaging.2014.01.014
41. Cai L, Fritz D, Stefanovic L, Stefanovic B. Binding of LARP6 to the Conserved 5′ Stem-Loop Regulates Translation of mRNAs Encoding Type I Collagen. J Mol Biol [Internet]. 2010;395(2):309–26. Available from: http://dx.doi.org/10.1016/j.jmb.2009.11.020
42. Zhang Y, Stefanovic B. LARP6 meets collagen mRNA: Specific regulation of type I collagen expression. Int J Mol Sci. 2016;17(3).
43. Glenn HL, Wang Z, Schwartz LM. Acheron, a Lupus antigen family member, regulates integrin expression, adhesion, and motility in differentiating myoblasts. Am J Physiol Physiol. 2009;298(1):C46–55.
44. Stefanovic B, Manojlovic Z, Vied C, Badger CD, Stefanovic L. Discovery and evaluation

of inhibitor of LARP6 as specific antifibrotic compound. Sci Rep. 2019;9(1):1–15.
45. Sukhanov S, Higashi Y, Shai S-Y, Snarski P, Danchuk S, D'Ambra V, et al. SM22α (Smooth Muscle Protein 22-α) Promoter-Driven IGF1R (Insulin-Like Growth Factor 1 Receptor) Deficiency Promotes Atherosclerosis. Arterioscler Thromb Vasc Biol [Internet]. 2018 Oct 1;38(10):2306–17. Available from: https://doi.org/10.1161/ATVBAHA.118.311134
46. Danchuk S, Delafontaine P, Higashi Y. Mir-1976 Downregulation and Phosphorylation of LARP6 are Associated with Insulin-like Growth Factor-1 (IGF1)-induced Collagen Upregulation in Vascular Smooth Muscle Cells. FASEB J [Internet]. 2019 Apr 1;33(1_supplement):522.9-522.9. Available from: https://www.fasebj.org/doi/abs/10.1096/fasebj.2019.33.1_supplement.522.9
47. Higashi Y, Danchuk S, Sukhanov S, Blackstock C, Li Z, Snarski P, et al. Abstract 13854: Micro-RNA Regulation of Collagen Production by Vascular Smooth Muscle Cells Mediated by La Ribonucleoprotein Domain Family Member 6: Potential Mechanisms Underlying Stable Phenotype of Atherosclerotic Plaque by Insulin-like Growth Factor 1. Circulation [Internet]. 2016 Nov 11;134(suppl_1):A13854–A13854. Available from: https://doi.org/10.1161/circ.134.suppl_1.13854
48. Assimes TL, Lee IT, Juang JM, Guo X, Wang TD, Kim ET, et al. Genetics of Coronary Artery Disease in Taiwan: A Cardiometabochip Study by the Taichi Consortium. PLoS One. 2016;11(3):e0138014.
49. Strawbridge RJ, Dupuis J, Prokopenko I, Barker A, Ahlqvist E, Rybin D, et al. Genome-wide association identifies nine common variants associated with fasting proinsulin levels and provides new insights into the pathophysiology of type 2 diabetes. Diabetes. 2011;60(10):2624–34.
50. Strawbridge RJ, Silveira A, Hoed M den, Gustafsson S, Luan J, Rybin D, et al. Identification of a novel proinsulin-associated SNP and demonstration that proinsulin is unlikely to be a causal factor in subclinical vascular remodelling using Mendelian randomisation. Atherosclerosis. 2017;266:196–204.
51. Fermin DR, Barac A, Lee S, Polster SP, Hannenhalli S, Bergemann TL, et al. Sex and age dimorphism of myocardial gene expression in nonischemic human heart failure. Circ Cardiovasc Genet. 2008;1(2):117–25.
52. Hoffmann P, Feige JJ, Alfaidy N. Expression and oxygen regulation of endocrine gland-derived vascular endothelial growth factor/prokineticin-1 and its receptors in human placenta during early pregnancy. Endocrinology. 2006;147(4):1675–84.
53. Alfaidy N, Hoffmann P, Boufettal H, Samouh N, Aboussaouira T, Benharouga M, et al. The Multiple Roles of EG-VEGF/PROK1 in Normal and Pathological Placental Angiogenesis. Biomed Res Int. 2014;2014:1–10.
54. Kisliouk T, Levy N, Hurwitz A, Meidan R. Presence and regulation of endocrine gland vascular endothelial growth factor/prokineticin-1 and its receptors in ovarian cells. J Clin Endocrinol Metab. 2003;88(8):3700–7.
55. LeCouter J, Kowalski J, Foster J, Hass P, Zhang Z, Dillard-Telm L, et al. Identification of an angiogenic mitogen selective for endocrine gland endothelium. Nature [Internet]. 2001;412(6850):877–84. Available from: https://doi.org/10.1038/35091000
56. Corlan AS, Cîmpean AM, Jitariu AA, Melnic E, Raica M. Endocrine Gland-Derived Vascular Endothelial Growth Factor/Prokineticin-1 in Cancer Development and Tumor Angiogenesis. Int J Endocrinol. 2017;2017.
57. Samson M, Peale F V., Frantz G, Rioux-Leclercq N, Rajpert-De Meyts E, Ferrara N.

Human endocrine gland-derived vascular endothelial growth factor: Expression early in development and in Leydig cell tumors suggests roles in normal and pathological testis angiogenesis. J Clin Endocrinol Metab. 2004;89(8):4078–88.
58. Mayanagi T, Sobue K. Diversification of caldesmon-linked actin cytoskeleton in cell motility. Cell Adhes Migr. 2011;5(2):150–9.
59. Tan X, Zhang X, Pan L, Tian X, Dong P. Identification of Key Pathways and Genes in Advanced Coronary Atherosclerosis Using Bioinformatics Analysis. Biomed Res Int. 2017;2017:1–12.
60. Wan L, Huang J, Ni H, Yu G. Screening key genes for abdominal aortic aneurysm based on gene expression omnibus dataset. 2018;1–13.
61. Goikuria H, Freijo M, Vega Manrique R, Sastre M, Elizagaray E, Lorenzo A, et al. Characterization of Carotid Smooth Muscle Cells during Phenotypic Transition. Cells. 2018;7(3):23.
62. Trillhaase A, Haferkamp U, Rangnau A, Märtens M. Di ff erentiation of human iPSCs into VSMCs and generation of VSMC-derived calcifying vascular cells. Stem Cell Res [Internet]. 2018;31(July):62–70. Available from: https://doi.org/10.1016/j.scr.2018.07.008
63. Zaina S, Heyn H, Carmona FJ, Varol N, Sayols S, Condom E, et al. DNA methylation map of human atherosclerosis. Circ Cardiovasc Genet. 2014;7(5):692–700.
64. Nazarenko MS, Markov A V., Lebedev IN, Freidin MB, Sleptcov AA, Koroleva IA, et al. A comparison of genome-wide DNA methylation patterns between different vascular tissues from patients with coronary heart disease. PLoS One. 2015;10(4):1–15.
65. Fernández-Sanlés A, Sayols-Baixeras S, Subirana I, Degano IR, Elosua R. Association between DNA methylation and coronary heart disease or other atherosclerotic events: a systematic review. Atherosclerosis. 2017;263:325–33.
66. Kichaev G, Bhatia G, Loh P, Gazal S, Burch K, Freund MK, et al. Leveraging Polygenic Functional Enrichment to Improve GWAS Power. Am J Hum Genet [Internet]. 2019;104(1):65–75. Available from: https://doi.org/10.1016/j.ajhg.2018.11.008
67. Li Q, Wineinger NE, Fu D, Libiger O, Alphs L, Savitz A, et al. Genome-wide association study of paliperidone efficacy. Pharmacogenet Genomics. 2017;(27):7–18.
68. Middeldorp CM, Hammerschlag AR, Ouwens KG, Groen-Blokhuis MM, Pourcain B St., Greven CU, et al. A Genome-Wide Association Meta-Analysis of Attention-De fi cit/Hyperactivity Disorder Symptoms in Population-Based Pediatric Cohorts. J Am Acad Child Adolesc Psychiatry. 2019;55(10):896–905.
69. Maguire LH, Handelman SK, Du X, Chen Y, Pers TH, Speliotes EK. Genome-wide association analyses identify 39 new susceptibility loci for diverticular disease. Nat Genet [Internet]. 2018;50(10):1359–1365. Available from: http://dx.doi.org/10.1038/s41588-018-0203-z
70. Vuorio T, Ylä-Herttuala E, Laakkonen JP, Laidinen S, Liimatainen T, Ylä-Herttuala S. Downregulation of VEGFR3 signaling alters cardiac lymphatic vessel organization and leads to a higher mortality after acute myocardial infarction. Sci Rep. 2018;8(1):1–13.
71. Dumont DJ, Jussila L, Taipale J, Lymboussaki A, Mustonen T, Pajusola K, et al. Cardiovascular Failure in Mouse Embryos Deficient in VEGF Receptor-3. Science (80-). 1998;282(October):946–50.
72. Schmeisser A, Christoph M, Augstein A, Marquetant R, Kasper M, Braun-Dullaeus RC, et al. Apoptosis of human macrophages by Flt-4 signaling: Implications for atherosclerotic plaque pathology. Cardiovasc Res. 2006;71(4):774–84.

73. Baeyens N, Nicoli S, Coon BG, Ross TD, Van den Dries K, Han J, et al. Vascular remodeling is governed by a VEGFR3-dependent fluid shear stress set point. Elife. 2015;4:1–16.
74. Tuenter A, Selwaness M, Lorza AA, Schuurbiers JCH, Speelman L, Vernooij MW, et al. High shear stress relates to intraplaque haemorrhage in asymptomatic carotid plaques. Atherosclerosis [Internet]. 2016;251:348–54. Available from: http://dx.doi.org/10.1016/j.atherosclerosis.2016.05.018
75. Camaré C, Pucelle M, Nègre-Salvayre A, Salvayre R. Angiogenesis in the atherosclerotic plaque. Redox Biol [Internet]. 2017;12(January):18–34. Available from: http://dx.doi.org/10.1016/j.redox.2017.01.007
76. Deng C, Tang S, Huang X, Gao J, Tian J. Identi fi cation of three novel loci of ALDH2 Gene for Serum Folate levels in a Male Chinese Population by Genome-Wide Association Study. Gene [Internet]. 2018;674(June):121–6. Available from: https://doi.org/10.1016/j.gene.2018.06.080

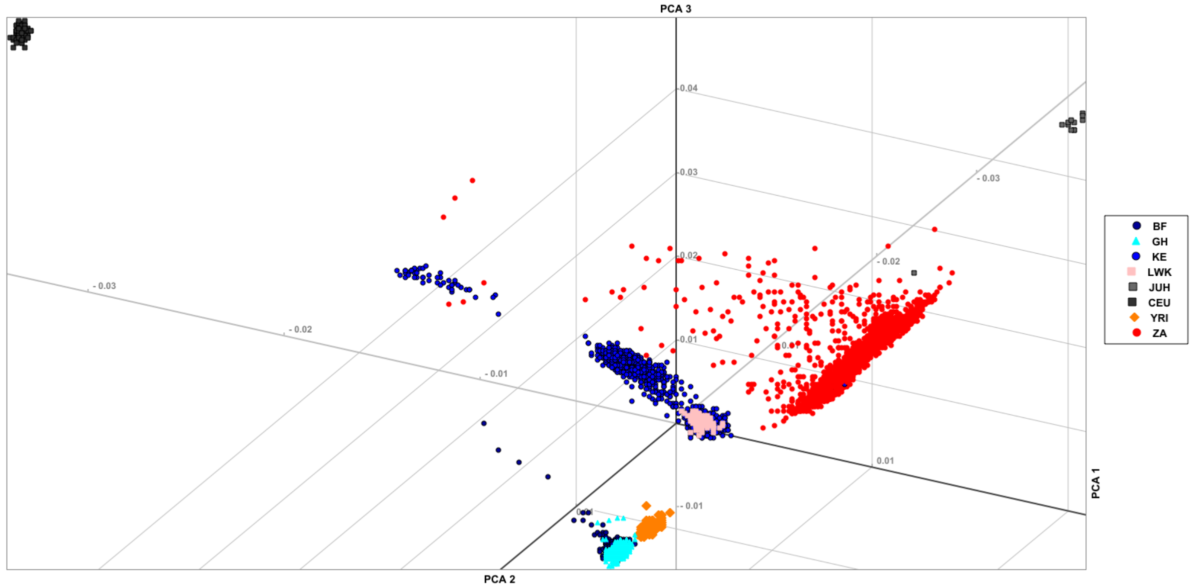

Supplementary Figure 1: Principal Component Analysis (PCA) plots showing population sub-structure in the AWI-Gen data and compared to data from selected populations from the 1000GP.

Each dot represents a participant and 1,729,661 SNPs were used in the analysis. AWI-Gen data - BF: Burkina Faso; GH: Ghana; KE: Kenya; ZA: South Africa.

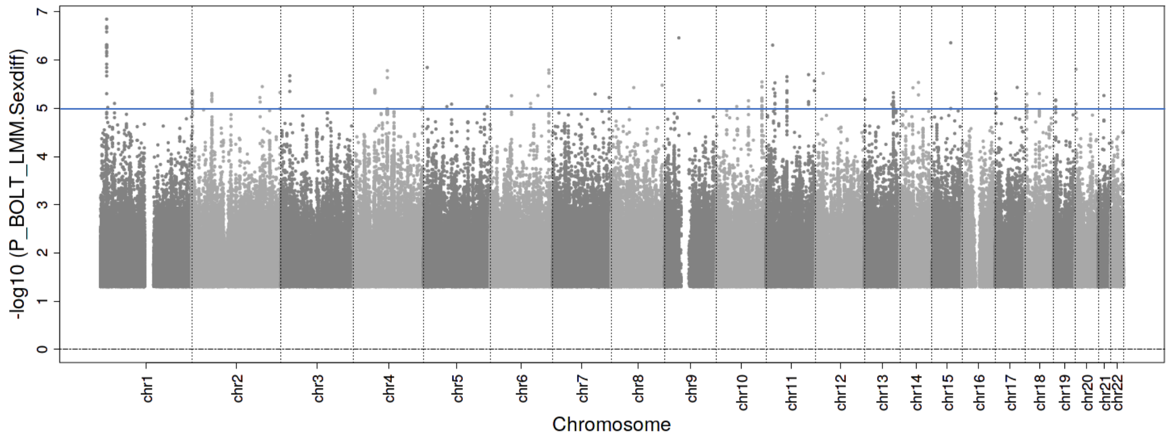

Supplementary Figure 2: Manhattan plot showing the –log10-transformed two-tailed P-value of each SNP for test of difference between female and male associations for each SNP.

The blue line indicates the threshold for suggestive association ($p < 1\times10^{-5}$). Each SNP from the GWAS for Mean Max cIMT on the Y axis and base-pair positions along the chromosomes on the X axis. The blue line indicates Bonferroni-corrected genome-wide significance (p < 1E-08); the blue line indicates the threshold for suggestive association (p < 1E-05).

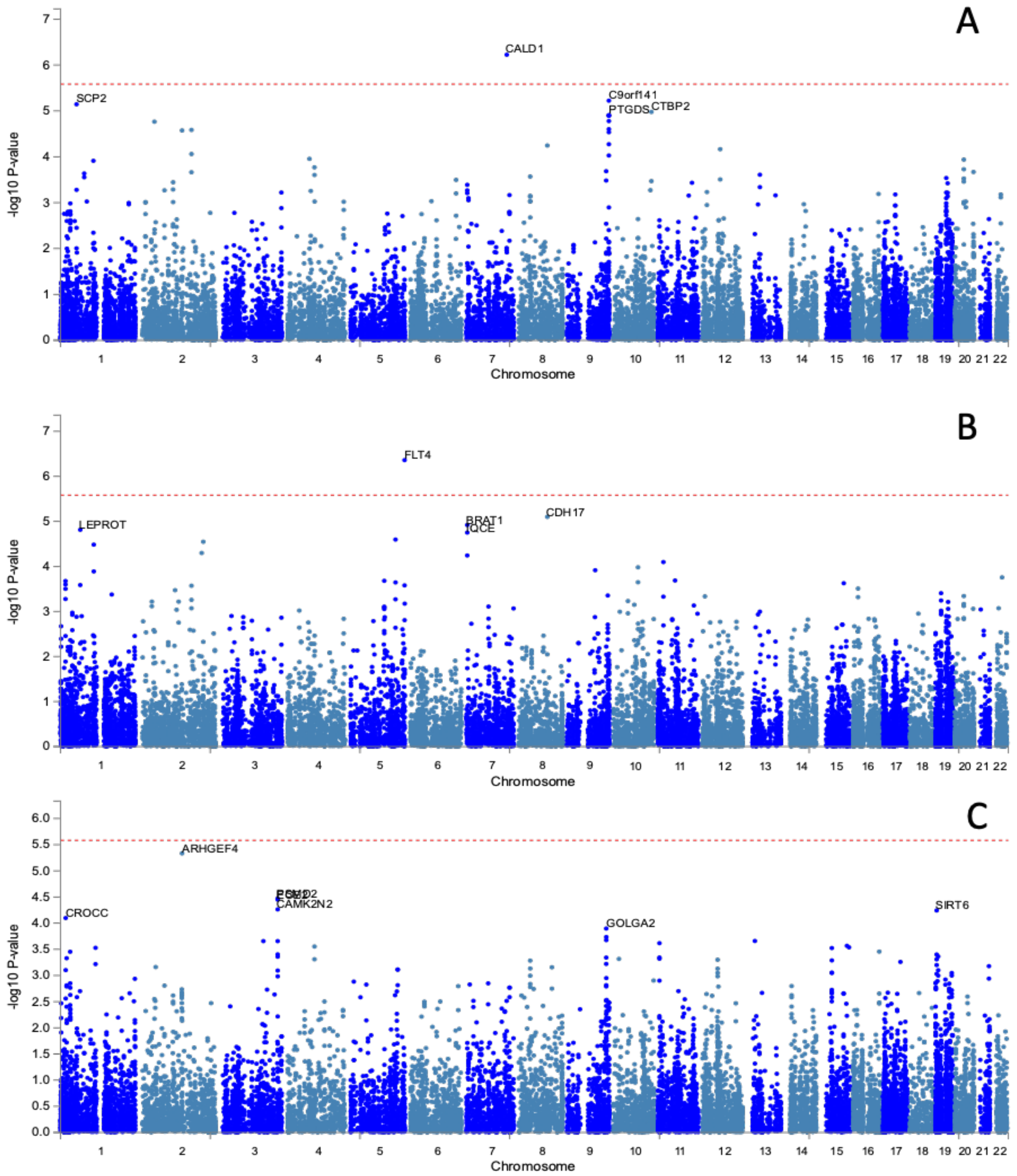

Supplementary Figure 3: Manhattan plots for the gene-based test as computed by MAGMA based on our summary statistics.

Input SNPs were mapped to 19152 protein coding genes. Genome wide significance (red dashed line in the plot) was defined at P = 0.05/19152 = 2.611e-6. (A) combined dataset. (B) Female-specific. (C) Male-specific

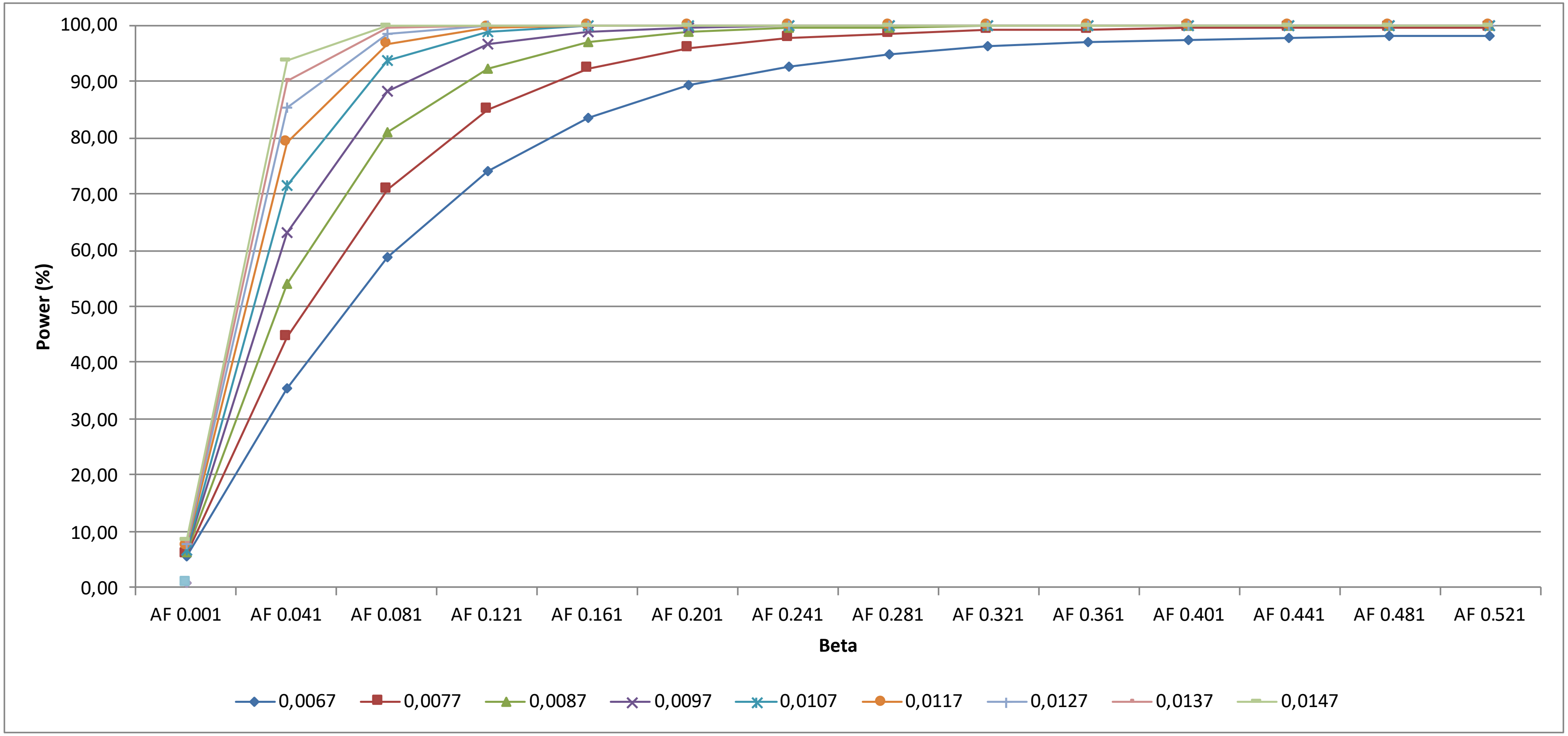

Supplementary Figure 4: Estimation of power for the GWAS of cIMT (as a quantitative trait) considering genetic effect β ranging from 0.0067 mm to 0.156 mm.
