# Supplementary material for: Genetic associations with carotid intima-media thickness link to atherosclerosis with sex-specific effects in sub-Saharan Africans
Source: Nat Commun. 2022 Feb 14;13:855. doi: 10.1038/s41467-022-28276-x (PMC8844072; doi:10.1038/s41467-022-28276-x)
Supplement: Supplementary file 3 — Description of Additional Supplementary Files [file 41467_2022_28276_MOESM3_ESM.pdf]

Supplementary Data 1: Descriptive characteristics of sample stratified by gender (men and women) and by regions (West, South and East)

Supplementary Data 2: Analysis of top SNPs variants in the combined analysis by regions (West, South and East)

Supplementary Data 3: Summary of SNPs with p-value <10-4 in at least one of the analysis

Supplementary Data 4: Selected SNPs (p<1E-06) for the sex-difference test

Supplementary Data 5: Summary of look-up from previously reported variants for cIMT

Supplementary Data 6: Summary of regions indicative of local replication from previously reported loci for cIMT

Supplementary Data 7: Look-up in Gwas Catalog for all SNPs with p-value <10-4 in at least one of the analysis

Supplementary Data 8a: Functional annotation for selected SNPs associated (p<1E-05) with cIMT in the combined analysis

Supplementary Data 8b: Functional annotation for selected SNPs associated (p<1E-05) with cIMT in the Female analysis

Supplementary Data 8c: Functional annotation for selected SNPs associated (p<1E-05) with cIMT in the Male analysis

Supplementary Data 9a: Genes implicated by positional, eQTL, or chromatin interaction mapping of SNPs associated with cIMT in the combined analysis

Supplementary Data 9b: Genes implicated by positional, eQTL, or chromatin interaction mapping of SNPs associated with cIMT in the Female analysis

Supplementary Data 9c: Genes implicated by positional, eQTL, or chromatin interaction mapping of SNPs associated with cIMT in the Male analysis

Supplementary Data 10a: Gene-sets with significant enrichment for genes associated with gene-smoking interaction for cIMT based on positional, eQTL, or chromatin interaction of suggestive SNPs (p<1E-05) with cIMT in the combined analysis.

Supplementary Data 10b: Gene-sets with significant enrichment for genes associated with gene-smoking interaction for cIMT based on positional, eQTL, or chromatin interaction of suggestive SNPs (p<1E-05) with cIMT in the female analysis.

Supplementary Data10c: Gene-sets with significant enrichment for genes associated with gene-smoking interaction for cIMT based on positional, eQTL, or chromatin interaction of suggestive SNPs (p<1E-05) with cIMT in the male analysis.

Supplementary Data 11: Post-hoc power calculations based on Gwas significant results
