# Supplementary material for: Genetic associations with carotid intima-media thickness link to atherosclerosis with sex-specific effects in sub-Saharan Africans
Source: Nat Commun. 2022 Feb 14;13:855. doi: 10.1038/s41467-022-28276-x (PMC8844072; doi:10.1038/s41467-022-28276-x)
Supplement: Supplementary file 21 — Reporting Summary [file 41467_2022_28276_MOESM21_ESM.pdf]

Corresponding author(s): Palwende Romuald Boua, Michele Ramsay

Last updated by author(s): Dec 13, 2021

Reporting Summary

Nature Research wishes to improve the reproducibility of the work that we publish. This form provides structure for consistency and transparency in reporting. For further information on Nature Research policies, see our Editorial Policies and the Editorial Policy Checklist.

Statistics

For all statistical analyses, confirm that the following items are present in the figure legend, table legend, main text, or Methods section.

n/a	Confirmed	
☐	☒	The exact sample size (*n*) for each experimental group/condition, given as a discrete number and unit of measurement
☒	☐	A statement on whether measurements were taken from distinct samples or whether the same sample was measured repeatedly
☐	☒	The statistical test(s) used AND whether they are one- or two-sided<br>*Only common tests should be described solely by name; describe more complex techniques in the Methods section.*
☐	☒	A description of all covariates tested
☐	☒	A description of any assumptions or corrections, such as tests of normality and adjustment for multiple comparisons
☐	☒	A full description of the statistical parameters including central tendency (e.g. means) or other basic estimates (e.g. regression coefficient) AND variation (e.g. standard deviation) or associated estimates of uncertainty (e.g. confidence intervals)
☐	☒	For null hypothesis testing, the test statistic (e.g. *F*, *t*, *r*) with confidence intervals, effect sizes, degrees of freedom and *P* value noted<br>*Give P values as exact values whenever suitable.*
☒	☐	For Bayesian analysis, information on the choice of priors and Markov chain Monte Carlo settings
☒	☐	For hierarchical and complex designs, identification of the appropriate level for tests and full reporting of outcomes
☒	☐	Estimates of effect sizes (e.g. Cohen's *d*, Pearson's *r*), indicating how they were calculated

Our web collection on statistics for biologists contains articles on many of the points above.

Software and code

Policy information about availability of computer code

Data collection	NA
Data analysis	EAGLE2;R v3.6.0; BOLT-LMM v2.3.1; http://github.com/h3abionet/h3agwas/; EasyStrata v8.6; Quanto (Version 1.2.4); http://fuma.ctglab.nl/;

For manuscripts utilizing custom algorithms or software that are central to the research but not yet described in published literature, software must be made available to editors and reviewers. We strongly encourage code deposition in a community repository (e.g. GitHub). See the Nature Research guidelines for submitting code & software for further information.

Data

Policy information about availability of data

All manuscripts must include a data availability statement. This statement should provide the following information, where applicable:

- Accession codes, unique identifiers, or web links for publicly available datasets
- A list of figures that have associated raw data
- A description of any restrictions on data availability

The processed data generated in this study are provided in the Supplementary Information. The AWI-Gen data used in this study are available to interested researchers through EGA, subject to controlled access review by the Data and Biospecimen Access Committee of the H3Africa Consortium. AWI-Gen (study ID: EGA00001002482) phenotype dataset is available EGAD00001006425 [https://ega-archive.org/datasets/EGAD00001006425]. AWI-Gen genotype dataset accession number: EGAD00010001996 [https://ega-archive.org/datasets/EGAD00010001996]. GWAS Catalog (https://www.ebi.ac.uk/gwas/).

Field-specific reporting

Please select the one below that is the best fit for your research. If you are not sure, read the appropriate sections before making your selection.

- [x] Life sciences
- [ ] Behavioural & social sciences
- [ ] Ecological, evolutionary & environmental sciences

For a reference copy of the document with all sections, see nature.com/documents/nr-reporting-summary-flat.pdf

Life sciences study design

All studies must disclose on these points even when the disclosure is negative.

Sample size	The AWI-Gen study enrolled 10,776 participants between 40-60 years old. Participants with both good quality cIMT and genotyping data (n = 7894) were used for the GWAS analyses.
Data exclusions	Individuals with a missing SNP calling rate greater than 0.05 were removed. SNPs with a genotype missingness greater than 0.05, MAF less than 0.01 and Hardy-Weinberg equilibrium (HWE) P-value less than 0.0001 were removed. Nonautosomal and mitochondrial SNPs, and ambiguous SNPs that did not match the GRCh37 references alleles or strands were removed. After imputation, poorly imputed SNPs with info scores less than 0.6, MAF less 0.01, and HWE P-value less than 0.00001 were excluded. The final QC-ed imputed data had 13.98 M SNPs. Mannheim Consensus defining the use of cIMT in population-based studies has been applied to QC cIMT values.
Replication	Not Applicable. There are no African study to allow replication.
Randomization	Not Applicable. This is a population-based study.
Blinding	Not Applicable. The study design do not require blinding.

Reporting for specific materials, systems and methods

We require information from authors about some types of materials, experimental systems and methods used in many studies. Here, indicate whether each material, system or method listed is relevant to your study. If you are not sure if a list item applies to your research, read the appropriate section before selecting a response.

Materials & experimental systems

n/a	Involved in the study	
x		Antibodies
x		Eukaryotic cell lines
x		Palaeontology and archaeology
x		Animals and other organisms
	x	Human research participants
x		Clinical data
x		Dual use research of concern

Methods

n/a	Involved in the study	
x		ChIP-seq
x		Flow cytometry
x		MRI-based neuroimaging

Human research participants

Policy information about studies involving human research participants

Population characteristics	The participants for this study include 10,703 black African men and women recruited from two urban settings (Nairobi and Soweto) and four rural settings (Agincourt, Dikgale, Nanoro and Navrongo), aged 40 to 60 years. the study was a cross-sectional study. Analysis were performed on 7894 individuals (3963 women, 3931 men). Population characteristics including covariates are provided in Supplementary Data.
Recruitment	Participants from the six sites (detail provided in Ali et al., 2018), and meeting the inclusion criteria were recruited after providing a written informed consent.
Ethics oversight	This study received approval from the Human Research Ethics Committee (Medical), University of the Witwatersrand, South Africa (M121029 and M170880 for the main study, and M1706110 for the cIMT project). Additionally, each contributing center received approvals from their local Ethics Committees.

Note that full information on the approval of the study protocol must also be provided in the manuscript.
